# ^18^F-fluorodeoxyglucose uptake predicts PKM2 expression in lung adenocarcinoma

**DOI:** 10.18632/oncotarget.17377

**Published:** 2017-04-24

**Authors:** Ping Huang, Xiang Zhao, Weiyuan Xiao, Yuqi Dong, Guangyu Hu

**Affiliations:** ^1^ Department of Orthopaedics, Ren Ji Hospital, School of Medicine, Shanghai Jiao Tong University, Shanghai, China

**Keywords:** lung adenocarcinomas, PKM2, glycolysis, ^18^F-FDG uptake

## Abstract

Positron emission tomography (PET) with ^18^F-fluorodeoxyglucose (^18^F-FDG) is widely used in the management of lung adenocarcinoma. Pyruvate kinase M2 (PKM2) plays a key role in glycolysis. We therefore investigated whether PKM2 expression affects ^18^F-FDG uptake in a retrospective analysis of 76 patients who underwent ^18^F-FDG PET/computed tomography (CT) scans for staging before surgical resection. We found that PKM2 expression was higher in tumors than peritumoral tissue (p < 0.05). Patients with high PKM2 expression had reduced overall (p < 0.05) and disease-free (p < 0.05) survival as compared to those with low PKM2 expression. Comparison of the primary tumor maximum standardized uptake value (SUVmax) between patients with high and low PKM2 expression revealed that the SUVmax was higher in primary tumors with high PKM2 expression than low PKM2 expression (p < 0.05). Multivariate analysis confirmed the association between SUVmax and PKM2 expression (p < 0.05). PKM2 status was predicted with 81.6% accuracy when the SUVmax cutoff value of 6.4. Thus,^18^F-FDG PET/CT is predictive of the PKM2 status in lung adenocarcinoma patients and could aid in determining therapeutic strategies.

## INTRODUCTION

Lung cancer is one of the most common malignancies worldwide, among which lung adenocarcinoma is the most common histologic type [1]. Although there have been advances in the early diagnosis and treatment of lung cancer, the 5-year survival rate is still poor [2]. This may be explained by the high metastasis rate of lung cancer and resistance to chemotherapeutics [3]. Improving the survival rate of lung adenocarcinoma patients requires the identification of biomarkers that can better predict tumor behavior (e.g. progression or metastatic capability).

The Warburg effect, which is characterized by a high rate of glycolysis even in the presence of adequate oxygen, is a hallmark of cancer cell growth [4]. Glycolysis is closely associated with cellular proliferation and drug resistance in cancer cells, and treatments that target glucose metabolism represent a new strategy for cancer therapy. Pyruvate kinase M2 (PKM2) regulates the final rate-limiting step of glycolysis by converting phosphoenolpyruvate to adenosine diphosphate (ADP) resulting in the generation of pyruvate and adenosine triphosphate (ATP) [5]. Upregulation of PKM2 ensures efficient synthesis of intermediates for biosynthetic reactions and ATP generation. PKM2 is over-expressed in many malignant tumors and plays an important role in development and metastasis [6–8]. Recently, several studies have shown that targeted molecular therapies that modulate PKM2 activity can improve the efficacy of chemotherapeutics [9–13]. However, some PKM2-targeted therapies have been ineffective in experimental and clinical studies. Therefore, it is important to identify the clinical characteristics of patients that can predict PKM2 status and the efficacy of PKM2-targeted therapies.

^18^F-fluorodeoxyglucose positron emission tomo graphy/computed tomography (^18^F-FDG PET/CT) is a molecular imaging technique that is widely used for diagnosing, staging, re-staging, and monitoring treatment response in cancer patients [14, 15]. Because glucose is the major energy substrate in tumor cells (the Warburg effect), increased ^18^F-FDG uptake is an indicator of poor prognosis in many malignant tumors such as lung cancer [16]. ^18^F-FDG uptake in cancer cells is primarily dependent upon on glucose transporters and hexokinases, which are over-expressed in many malignant tumors. Although PKM2 plays a crucial role in the Warburg effect [17], the relationship between ^18^F-FDG accumulation and PKM2 expression has not been examined.

In this study, we examined PKM2 expression in patients with lung adenocarcinoma and evaluated the relationship between PKM2 expression and patient prognosis. We also investigated the relationship between PKM2 expression and ^18^F-FDG uptake to determine whether ^18^F-FDG uptake could be used to predict PKM2 status in lung adenocarcinoma patients. Finally, we examined whether glucose transporter-1 (GLUT1) and hexokinase 2 (HK2) expression was associated with ^18^F-FDG uptake, and assessed the correlation between PKM2 and GLUT1 and HK2 in lung adenocarcinomas. Our results suggest that ^18^F-FDG PET can be used to predict PKM2 status, and that ^18^F-FDG PET may help determine the therapeutic strategy for lung adenocarcinoma patients by predicting tumor response to PKM2-targeted therapies.

## RESULTS

### Patient characteristics

The clinicopathological characteristics of the 76 lung adenocarcinoma patients (34 men and 42 women) are shown in Table 1. The mean patient age was 64 years, and the age range was 38-85 years. The mean primary tumor maximum standardized uptake value (SUVmax) was 9.67 (range, 1-35.5). The mean tumor size was 3.1 cm (range, 1-9.5 cm). Lymph node metastasis was detected in 25 patients. Most patients had stage I disease (57.9%), followed by stage II (21.1%), stage III (15.8%), and stage IV disease (5.3%).

**Table 1:** PKM2 expression in paired tissue specimens from 76 patients with lung adenocarcinoma

Specimen	PKM2 expression		p-value
	High	Low	
Tumor tissue	41	35	p < 0.05
Peritumoral tissue	10	66	

### PKM2 expression is upregulated in lung adenocarcinoma patients

**Figure 1:**
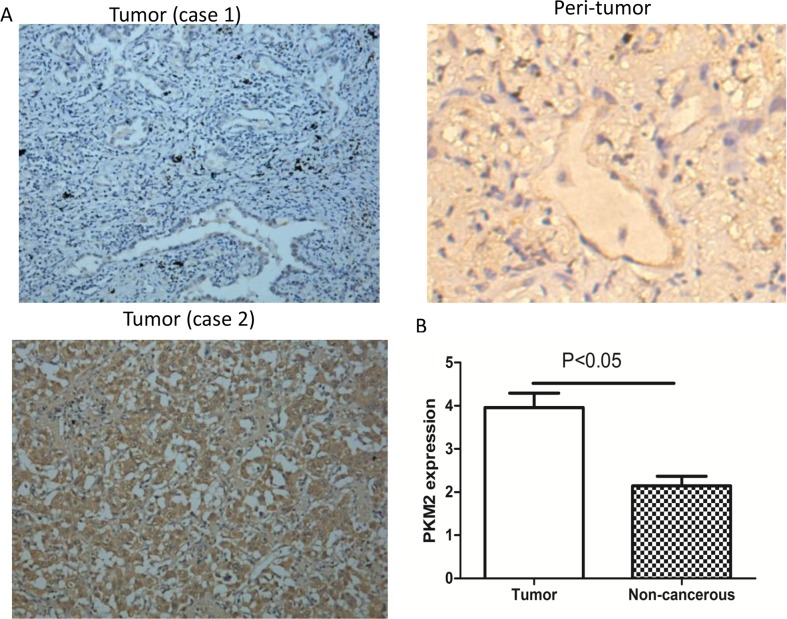
Immunohistochemical staining of PKM2 in lung adenocarcinoma specimens **(A)** Case 1: Tumor tissue with high PKM2 expression. Case 2: Tumor tissue with low PKM2 expression. Peritumoral tissues have low PKM2 expression (×200 magnification). **(B)** The mean PKM2 immunohistochemical staining score in lung adenocarcinoma tissue (3.96 ± 2.91) was significantly higher than that of matched peritumoral tissue (p < 0.05) (2.14 ± 1.94).

To understand the role of PKM2 in lung adenocarcinoma, we compared PKM2 expression between tumor and matched normal tissue specimens from the 76 patients. In the tumor cells, PKM2 expression was mainly detected in the cytoplasm and occasionally in the nucleus (Figure 1A). High PKM2 expression was observed in 53.9% (41/76) of tumor and 13.1% (10/76) of peritumoral tissues (Table 1). The mean PKM2 immunohistochemical staining score in the tumor tissues (3.96 ± 2.91) was significantly higher than that in the peritumoral tissues (2.14 ± 1.94) (Figure 1B). Based on the immunohistochemical analysis, the lung adenocarcinoma patients were classified into two groups: those with high (n = 41) and low (n = 35) PKM2 expression. The relationship between PKM2 expression and various patient clinicopathological parameters is illustrated in Table 2. PKM2 expression was correlated with tumor differentiation, lymph node metastasis, and tumor-node-metastasis (TNM) stage (p < 0.05; Table 2). However, there were no significant differences between PKM2 expression and age, sex, tumor size, or distant metastasis.

**Table 2:** Relationship between PKM2 expression and the clinicopathological characteristics of lung adenocarcinoma patients (n = 76)

Variable	PKM2 expression		p-value
	Low	High	
**Age (years)**			
< 60	11	12	0.838
≥ 60	24	29	
**Sex**			
Female	20	22	0.761
Male	15	19	
**Tumor size (cm)**			
≤ 3	32	31	0.068
> 3	3	10	
**Lymph node metastasis**			
Negative	28	23	0.027
Positive	7	18	
**Distant metastasis**			
Negative	35	37	0.058
Positive	0	4	
**Tumor differentiation**			
Well	11	3	0.017
Moderate	15	19	
Poor	9	19	
**TNM stage**			
I	26	18	0.025
II	4	12	
III	5	7	
IV	0	4	

### Survival analysis

**Figure 2:**
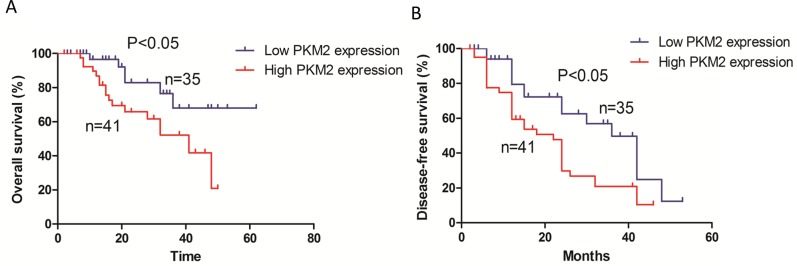
Kaplan-Meier survival analysis of 76 patients with lung adenocarcinoma **(A)** The overall survival rate in patients with high PKM2 expression was significantly lower than that of patients with low PKM2 expression. **(B)** The disease-free survival rate in patients with high PKM2 expression was significantly lower than that of patients with low PKM2 expression.

Kaplan-Meier survival curves indicated that patients with high PKM2 expression had reduced overall survival (p < 0.05) compared to those with low PKM2 expression (Figure 2A). In addition, patients with high PKM2 expression had reduced disease-free survival (p < 0.05) compared to those with low PKM2 expression (Figure 2B).

### The relationship between SUVmax and PKM2 expression

**Figure 3:**
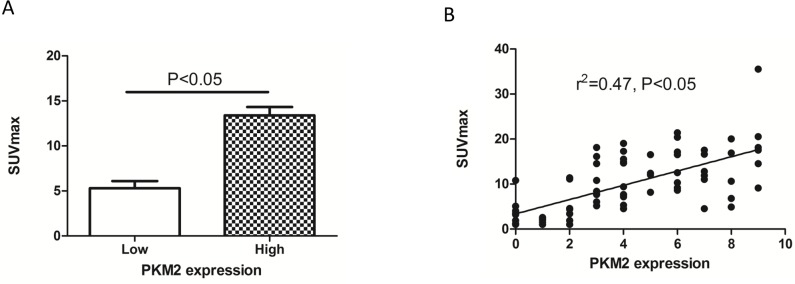
The relationship between PKM2 expression and SUVmax in lung adenocarcinoma **(A)** The SUVmax was significantly higher in patients with high (13.39 ± 6.002) compared to low PKM2 expression (5.31 ± 4.645) (p < 0.05). **(B)** The SUVmax and PKM2 expression are positively correlated (r^2^ = 0.47, p < 0.05).

**Figure 4:**
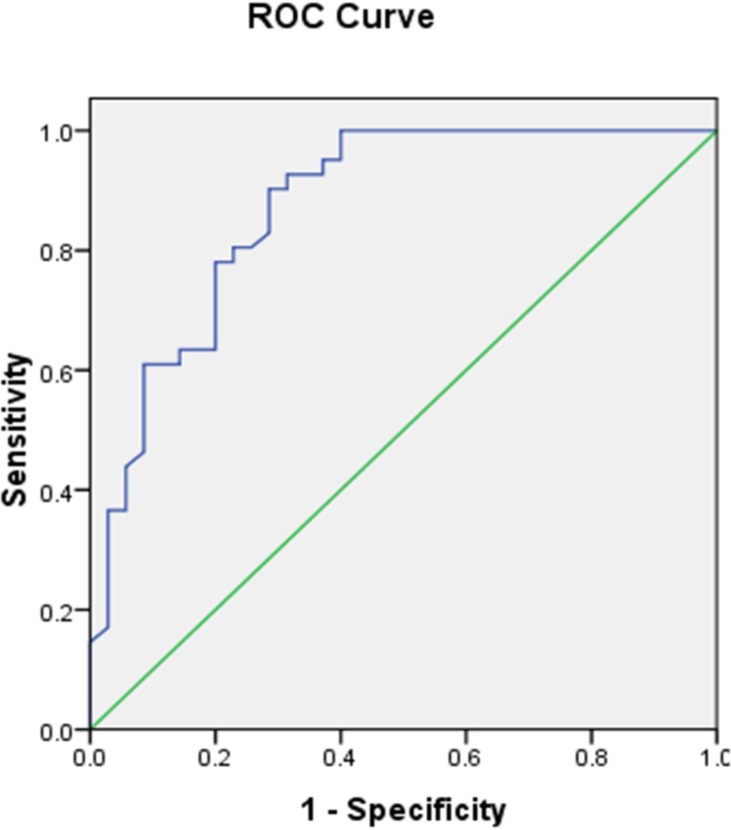
ROC curve of the SUVmax for predicting PKM2 expression The area under the ROC curve was 0.874 ± 0.041. When the threshold of the SUVmax was set at 6.4, the sensitivity and specificity were 90.2% and 71.4%, respectively.

We next assessed the relationship between SUVmax and PKM2 expression in lung adenocarcinomas. The SUVmax was significantly higher in tumors with high compared to low PKM2 expression (13.39 ± 6.002 vs. 5.31 ± 4.645; p < 0.0001) (Figure 3A). A positive correlation was observed between the SUVmax and PKM2 expression in lung adenocarcinomas (r^2^ = 0.47, p < 0.05; Figure 3B). We determined the SUVmax threshold for optimal differentiation between patients with high and low PKM2 expression. Receiver operating characteristic (ROC) curve analysis revealed that the highest accuracy (81.6%) for predicting PKM2 status was obtained with a SUVmax cutoff value of 6.4 (the area under the curve was 0.874 ± 0.041; Figure 4). The sensitivity and specificity of the SUVmax for predicting PKM2 expression were 90.2% (37/41) and 71.4% (25/35), respectively. These results suggested that the SUVmax of primary tumors could be used to predict PKM2 status in lung adenocarcinoma patients. Multivariate analysis was performed to exclude the possibility that the predictive value of SUVmax for PKM2 status. In the multivariate analysis, the SUVmax remained correlated with PKM2 expression in lung adenocarcinoma patients (Table 3).

**Table 3:** Multivariate analysis of PKM2 expression in 76 patients with lung adenocarcinoma

Parameter	Univariate analysis		p-value
	OR	95% CI	
Sex	2	0.446-5.268	0.497
Age (years)	1.079	0.267-4.357	0.910
Tumor size (cm)	1.084	0.192-6.127	0.927
Tumor differentiation	0.722	0.239-2.182	0.564
Lymph node metastasis	0.632	0.106-3.768	0.615
TNM stage	1.31	0.525-3.269	0.563
SUVmax	28.464	4.658-173.938	0.001

OR: Odds ratio; 95% CI: 95% Confidence interval

### The SUVmax is correlated with PKM2 and GLUT1 expression

**Figure 5:**
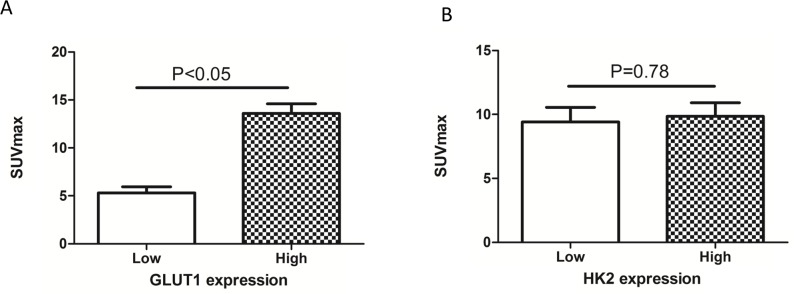
Relationship between SUVmax and GLUT1 and HK2 expression **(A)** The SUVmax was significantly higher in tumors with high (13.6 ± 6.3) compared to low GLUT1 expression (5.3 ± 3.9) (p < 0.05). **(B)** There was no significant difference in SUVmax between the high and low HK2 expression groups (p > 0.05).

We also assessed the relationship between the SUVmax and the expression of GLUT1 and HK2 using immunohistochemistry. The SUVmax was higher in tumors with high (13.6 ± 6.3) compared to low (5.3 ± 3.9; p < 0.05) GLUT1 expression (Figure 5A). However, there was no significant difference in the SUVmax between patients with high and low HK2 expression (Figure 5B). PKM2 and GLUT1 expression were correlated (r^2^ = 0.604, p < 0.05; Table 4), but PKM2 and HK2 expression were not (r^2^ = -0.064, p > 0.05; Table 4). These results suggest that PKM2 affects ^18^F-FDG uptake in non-small cell lung cancer, possibly through upregulation of GLUT1 expression.

**Table 4:** Relationship between PKM2 and GLUT1 and HK2 expression

		PKM2 expression		p-value
		Low	High	
GLUT1 expression	Low	28	8	< 0.05
	High	8	33	
HK2 expression	Low	14	21	> 0.05
	High	19	22	

## DISCUSSION

The Warburg effect confers a growth advantage to cancer cells by transforming glucose into substrates and ATP for tumor growth. PKM2 is an important metabolic enzyme responsible for the Warburg effect. It is over-expressed in various cancers including tongue cancer, pancreatic cancer, and hepatocellular carcinoma [18–20]. Our results suggest that PKM2 expression is significantly higher in lung cancer compared to noncancerous tissue. Interestingly, PKM2 expression was correlated with tumor differentiation, lymph node metastasis, and TNM stage. Our data indicate that PKM2 is over-expressed in a significant fraction of lung adenocarcinomas suggesting that PKM2 might play a critical role in tumor development and serve as a novel therapeutic target in lung adenocarcinoma.

Given that PKM2 is a potential therapeutic target, it is important to identify the clinical characteristics of patients that can predict PKM2 status or the efficacy of therapeutics that target PKM2. We evaluated PKM2 expression in patient tissue samples using immunohistochemistry. However, this approach involves an invasive procedure and is limited by the availability of tumor tissue. The development of noninvasive techniques such as PET/CT for predicting tumor molecular profiles could help overcome this limitation. We analyzed the association between PKM2 expression and ^18^F-FDG uptake in lung adenocarcinoma patients and determined that the SUVmax was significantly higher in lung adenocarcinomas with high compared to low PKM2 expression.

*In vitro* assays using cancer cell lines have indicated that PKM2 knockdown inhibits glucose uptake and lactate production [5, 21]. Consistent with these results, we observed a 1.5-fold increase in the SUVmax in primary tumors with high compared to low PKM2 expression (p < 0.05). ROC analysis indicated that ^18^F-FDG uptake could predict PKM2 expression in lung adenocarcinoma patients. Multivariate analysis demonstrated that the SUVmax of the primary tumor was correlated with PKM2 expression. These results suggest that ^18^F-FDG PET/CT scans could be useful for predicting PKM2 status in lung adenocarcinoma patients. Since many PKM2 inhibitors are in development for the treatment of lung adenocarcinoma patients with high PKM2 expression, noninvasive methods (e.g. molecular imaging) that can predict PKM2 status and be used to monitor the effects of PKM2-targeted therapeutics are of great clinical relevance.

Previous studies have demonstrated that PKM2 can increase GLUT1 expression *in vitro* [22, 23]. Consistent with these results, we observed a positive correlation between PKM2 and GLUT1 expression in lung adenocarcinoma patient tissue samples. The SUVmax was significantly higher in tumors with high compared to low GLUT1 expression. These data suggest that PKM2 affects ^18^F-FDG uptake in lung adenocarcinoma, possibly by upregulating GLUT1 expression.

Our study was limited in part by its retrospective design and the small sample size. Although PET/CT may have moderate diagnostic performance, it is not possible to establish a cutoff value for SUVmax in the clinical setting. Therefore, PET/CT cannot replace conventional diagnostic methods. Nonetheless, our results may be relevant for developing noninvasive strategies for predicting PKM2 expression or for monitoring the therapeutic effects of PKM2 inhibitors in lung adenocarcinoma patients.

## CONCLUSION

Lung adenocarcinoma patients with high PKM2 expression show increased ^18^F-FDG uptake on PET/CT. Thus, PET/CT may be a complementary tool for assessing the molecular profiles of lung adenocarcinomas and for predicting PKM2 expression. It could be particularly useful in cases where tumor tissue is not available or for monitoring the effects of PKM2-targeted therapeutics. PKM2 expression is positively correlated with GLUT1 expression, and PKM2 may increase ^18^F-FDG uptake through upregulation of GLUT1 expression. Additional large, prospective studies are needed to confirm our results and determine whether PET/CT can be used in lung adenocarcinoma patients to predict PKM2 status. This will aid in clinical decision-making regarding therapeutic strategies for lung adenocarcinoma.

## MATERIALS AND METHODS

### Study population

Seventy-six patients (34 men and 42 women; age range, 38-85 years; mean age, 64.3 years) with lung adenocarcinoma were included in the study. All patients underwent ^18^F-FDG PET/CT before surgery at the Shanghai Jiaotong University-affiliated Ren Ji Hospital between January 2012 and June 2016. The diagnosis of lung adenocarcinoma was confirmed by pathological examination of tumor specimens. The complete case information including age, sex, tumor differentiation and TNM stage, survival, and recurrence was available. The Institutional Review Board of the Shanghai Jiao Tong University-affiliated Ren Ji Hospital approved this study, and all patients provided written informed consent.

### PET/CT imaging

Images were acquired using a combined PET/CT device (Biograph mCT; Siemens). The patients fasted for at least 4 h before intravenous injection of ^18^F-FDG, and acquisition of whole-body PET images started 50 min after the injection. CT data were used for attenuation correction and PET image datasets were reconstructed iteratively. For semi-quantitative analysis of ^18^F-FDG uptake, regions of interest were manually defined on transaxial tomograms. The SUVmax was calculated as follows: maximum pixel value with the decay-corrected region of interest activity (MBq/kg)/(injected dose [MBq]/body weight [kg]). Two experienced nuclear medicine physicians evaluated the PET images.

### Immunohistochemistry

Tumor tissues were fixed in 10% formalin and embedded in paraffin. Formalin-fixed paraffin-embedded sections (5 μm) were stained with antibodies against GLUT1, HK2, and PKM2. All primary antibodies were purchased from Proteintech (Proteintech Group, USA). Two pathologists independently scored the immunohistochemistry results according to intensity and percentage of PKM2-, GLUT1-, or HK2-positive cells.

### Statistical analysis

PKM2 expression in tumor compared to matched non-cancerous tissue was analyzed with a parametric test. The association between PKM2 expression and various clinical parameters of lung adenocarcinoma patients was assessed using chi-square tests. Kaplan-Meier analysis was used to evaluate the association between PKM2 expression and lung adenocarcinoma patient outcomes. The relationships between PKM2 expression and GLUT1 or HK2 expression were analyzed using Pearson correlation coefficients. For all tests, the significance level was set at p < 0.05. Statistical analyses were performed using SPSS 13.0 (SPSS Inc., Chicago, IL, USA).
